# Synergistic Effects of Simvastatin and Irinotecan against Colon Cancer Cells with or without Irinotecan Resistance

**DOI:** 10.1155/2016/7891374

**Published:** 2016-02-04

**Authors:** Hyun Joo Jang, Eun Mi Hong, Juah Jang, Jung Eun Choi, Se Woo Park, Hyun Woo Byun, Dong Hee Koh, Min Ho Choi, Sea Hyub Kae, Jin Lee

**Affiliations:** ^1^Division of Gastroenterology, Department of Internal Medicine, Dongtan Sacred Heart Hospital, Hallym University School of Medicine, Gyeonggi-do 18450, Republic of Korea; ^2^Division of Gastroenterology, Department of Internal Medicine, Hangang Sacred Heart Hospital, Hallym University School of Medicine, Seoul, Republic of Korea

## Abstract

*Aims*. We here investigated whether the combination of simvastatin and irinotecan could induce the synergistic effect on colon cancer cells with or without resistance to irinotecan.* Methods*. We investigated cell proliferation assay and assessed cell death detection ELISA and caspase-3 activity assay of various concentrations of simvastatin and irinotecan to evaluate the efficacy of drug combination on colon cancer cells with or without irinotecan resistance.* Results*. The IC_50_ values of simvastatin alone and irinotecan alone were 115.4 ± 0.14 *μ*M (*r* = 0.98) and 62.5 ± 0.18 *μ*M (*r* = 0.98) in HT-29 cells without resistance to irinotecan. The IC_50_ values of these two drugs were 221.9 ± 0.22 *μ*M (*r* = 0.98) and 195.9 ± 0.16 *μ*M (*r* = 0.99), respectively, in HT-29 cell with resistance to irinotecan. The results of combinations of the various concentrations of two drugs showed that combined treatment with irinotecan and simvastatin more efficiently suppressed cell proliferation of HT-29 cells even with resistance to irinotecan as well as without resistance. Furthermore, the combination of simvastatin and irinotecan at 2 : 1 molar ratio showed the best synergistic interaction.* Conclusion*. Simvastatin could act synergistically with irinotecan to overcome irinotecan resistance of colon cancer.

## 1. Introduction

Colorectal cancer (CRC) is one of the most common cancers, and it is an important cause of cancer-related mortality worldwide [1, 2]. The incidence of CRC, moreover, shows also rapidly increasing tendency in Korea [3, 4]. Although improved oncologic therapeutic progress and advanced treatment regimen have positively influenced CRC prognostic outcomes, the five-year survival rate remains low in advanced or metastatic CRC [5, 6]. Anticancer drugs such as irinotecan and 5-fluorouracil (5-FU) have been administered, often with curative therapy, to eliminate circulating cancer cells. 5-FU inhibits thymidylate synthase, an enzyme for the synthesis of pyrimidines in order to replicate DNA [7]. Irinotecan is a topoisomerase I inhibitor which results in stabilization of the cleavable complex, breakage of DNA strands, failure of DNA replication, and ultimately cell death [8–10]. Nevertheless, overall response rates of CRC patients treated with these cytotoxic drugs remain less than 40% when these drugs are used as monotherapies [11, 12]. Therefore, it is necessary to develop other new drugs or new combinations of drugs, to provide other therapeutic options to eradicate colon cancer cells and improve the survival of colon cancer patients.

3-Hydroxy-3-methyl glutaryl-CoA reductase inhibitors, known as statins, not only reduce serum cholesterol and decrease the risk of cardiovascular and cerebrovascular events but reduce the risk of CRC [13–15]. Recently, statins have been studied for their pleiotropic effects including anti-inflammatory, antioxidant, and anticancer effects. Statins reduce serum cholesterol levels by competitively inhibiting HMG-CoA reductase, which is the rate-limiting enzyme in the mevalonate pathway. Mevalonate is involved in the synthesis of isoprenyl proteins, dolichol, and ubiquinone that play several important roles in cellular functions such as cell signaling, cell proliferation, cell growth, and respiration. Some recent studies showed that statins might be beneficial as anticancer drugs [16, 17]. Their antitumor effects may be due to inhibition of cell proliferation, promotion of apoptosis, inhibition of angiogenesis, and prevention of metastasis [18–21]. Statins might be a chemosensitizer of 5-FU to cancer cells [22]. We also had previously investigated that simvastatin induces the apoptosis of colon cancer cells through control of the expression of IGF-1R and IGF-1R signaling pathways.

We hypothesized that statin could synergistically act with irinotecan in CRC treatment, and the combination of a statin with irinotecan could help to overcome irinotecan resistance in colon cancer cells. In this study, we investigated the combination effect of a statin with irinotecan in CRC cells with or without irinotecan resistance.

## 2. Materials and Methods

### 2.1. Materials

Dulbecco's modified Eagle medium (DMEM), fetal bovine serum, trypsin/EDTA, and penicillin/streptomycin were from Gibco (Grand Island, NY, USA). Simvastatin was from Calbiochem (Gibbstown, NJ, USA). All other chemicals were bought from Sigma (St. Louis, MO, USA).

### 2.2. Cell Culture

Human HT-29 cells were cultured in DMEM with 4.5 g/L glucose and 2 mM glutamine added with 10% fetal bovine serum, 1.5 g/L sodium bicarbonate, 100 *μ*g/mL streptomycin, and 100 IU/mL penicillin. The medium was exchanged twice a week, and the cells were cultured in 37°C incubator with 5% CO_2_. When the cells were confluent (every 5 to 7 days), the cells were subcultured using trypsin (2.5 g/L) and EDTA (1 g/L). Experiments were carried out in serum-free medium (SFM) containing 0.1% bovine serum albumin (BSA, Sigma, St. Louis, MO, USA). The irinotecan-resistant HT-29 cells had been isolated from the HT-29 cells with gradually increasing the concentration of irinotecan [23]. At first, the HT-29 cells were cultured in the medium containing 1 nM irinotecan. The concentration of irinotecan in the medium was gradually increased every two weeks. Finally, the cells were incubated with the medium containing 16 nM irinotecan and then the irinotecan-resistant HT-29 cells were obtained eight months later. The cells were cultured in the irinotecan-free medium at least a week prior to any experiment.

### 2.3. MTT (3-[4,5-Dimethylthiazol-2-yl]-diphenyltetrazolium Bromide) Assay

Cell proliferation of HT-29 was measured by MTT assay. The cells were seeded at a density of 5 × 10^4^ cells/mL with a cultured medium in a 96-well plate. After incubation for 24 h, HT-29 cells were treated with various concentration of simvastatin or irinotecan in serum-free medium for 24 h. Then MTT (0.5 mg/mL) (Sigma, St. Louis, MO, USA) was added to each well and incubated for further 4 h at 37°C. After the medium has been removed, 100 *μ*L of dimethyl sulfoxide (DMSO) was added to each well by shaking the plate for 10 min. The optical density (OD) was evaluated by DTX 880 Multimode Detector (Beckman Coulter, Brea, CA, USA) at 570 nm. Each assay was performed in triplicate.

### 2.4. Drug Combination Analysis

The combination index (CI) was calculated by the computer software CalcuSyn (version 1.1.1, 1996, Biosoft, Cambridge, UK) based on the Chou-Talalay equation which takes into account both the shape of the dose-effect curve and potency (Dm or IC_50_). CI < 1 indicates synergism, CI = 1 additive effect, and CI > 1 antagonism. In order to calculate CI, we first had obtained the IC_50_ value of each agent and tested various combinations of these drugs in each colon cancer cell line. The IC_50_ values of each agent were determined by Prism 5 (GraphPad Software) from which concentrations were established for use in combination experiments. Drug interaction was assessed calculating the CI.

### 2.5. Cell Death Assay

Cell apoptosis was assessed by the detection of monooligonucleosomes (histone-associated DNA fragments) using an ELISA kit Cell Death Detection ELISA^plus^ kit (Roche Applied Science, Mannheim, Germany) according to the manufacturer's instructions. HT-29 cells were seeded in 96-well plate at a concentration of 1 × 10^4^ cells/well with the culture medium and incubated for twenty-four hours. Cells were treated with various concentrations of irinotecan or simvastatin for 48 h. After the medium has been removed, cells were treated with 100 *μ*L of lysis buffer for 30 min and centrifuged at 200 g at 4°C for 10 min. The supernatant (cell lysate solution) was placed in a well of streptavidin-coated plate supplied by the manufacturer. A mixture of anti-histone-biotin and anti-DNA-POD was treated to a cell lysate and incubated for 2 h. After the plate was washed, 100 *μ*L of ABTS (2,2′-azinobis-3-ethyl-benzothiazoline-6-sulfonic acid) substrate was added in each well of the plate for 20 min. The absorbance at 405 nm was checked with DTX 880 Multimode Detector (Beckman Coulter, Brea, CA, USA).

### 2.6. Caspase-3 Activity Assay

Caspase-3 activity assay (BioVision, Mountain View, CA, USA) was used to measure caspase-3 activity, according to the manufacturer's instructions. Cells were plated on 60 mm dishes in culture medium at a concentration of 2 × 10^6^ cells/mL and treated with various concentration of irinotecan or simvastatin for 48 h. Cells were washed with PBS and harvested with lysis buffer. Cells were maintained on ice for 10 minutes. Cell lysate was centrifuged at 4°C and 12,000 g and supernatant was transferred to a new tube and stored on ice. Protein contents were analyzed using the Bradford assay (Sigma, St. Louis, MO, USA). Assays were performed in 96-well plates by containing 90 *μ*g of protein in 50 *μ*L lysis buffer, and 5 *μ*L of 4 mM DEVD-pNA was added to the protein samples. The samples were incubated at 37°C for 2 h. Absorbance was measured at 405 nm using DTX 880 Multimode Detector (Beckman Coulter, Brea, CA, USA).

### 2.7. Statistical Analysis

Results from each experiment were expressed as the mean ± SD of three separate experiments. Data were analyzed by one-way analysis of variance (ANOVA), by Tukey's multiple comparison tests, and by Student's *t*-test using GraphPad Prism 4.0 software. *P* values < 0.05 were considered statistically significant.

## 3. Results

### 3.1. Establishment of Irinotecan-Resistant Cell Lines

To obtain irinotecan-resistant cell lines, HT-29 cells were treated to gradually increasing concentrations of irinotecan. We first obtained the HT-29 cells and selected as a reference one of the clones named HT-29. An irinotecan-resistant clone, named HT-29R, was acquired from the cell population growing in the medium containing 16 nM irinotecan.

The IC_50_ value of irinotecan or simvastatin in the respective cell line was estimated in order to confirm the irinotecan-resistant cell lines. While the IC_50_ values of simvastatin and irinotecan in HT-29 were 115.4 ± 0.14 *μ*M (*r* = 0.98) and 62.5 ± 0.18 *μ*M (*r* = 0.98), respectively, the IC_50_ values of those were 221.9 ± 0.22 *μ*M (*r* = 0.98) and 195.9 ± 0.16 *μ*M (*r* = 0.99) in HT-29R.

### 3.2. Effect of Simvastatin or Irinotecan as Single Agents in HT-29 and HT-29R Cells

**Figure 1:**
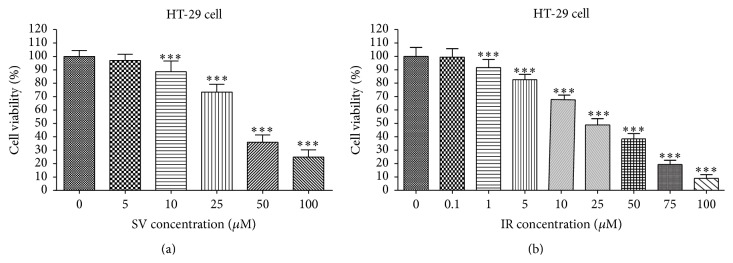
Effect of simvastatin or irinotecan alone on cell viability in HT-29 cell. Human colon cancer cells HT-29 were treated with serial concentrations of simvastatin (SV) and irinotecan (IR) for 48 h in 96-well plates, and cell viability was measured by MTT assay, respectively (a, b). The data are expressed as the means ± SD from three independent experiments. ^*∗∗∗*^ *P* < 0.001, compared to untreated control cells.

**Figure 2:**
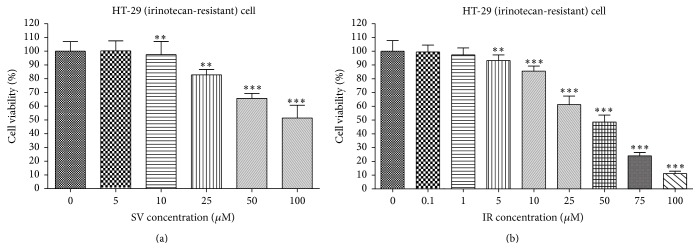
Effect of simvastatin or irinotecan alone on cell viability in irinotecan-resistant HT-29 cell. Human colon cancer cells HT-29 with irinotecan resistance were treated with serial concentrations of simvastatin (SV) and irinotecan (IR) for 48 h in 96-well plates, and cell viability was measured by MTT assay, respectively (a, b). The data are expressed as the means ± SD from three independent experiments. ^*∗∗∗*^ *P* < 0.001, compared to untreated control cells; ^*∗∗*^ *P* < 0.05, compared to untreated control cells.

As shown in Figures 1 and 2, treatment of simvastatin and irinotecan induced dose-dependent growth inhibition in both cell lines, with or without irinotecan resistance. We observed simvastatin and irinotecan lowered cell viability effectively depending on each drug concentration in both HT-29 (Figure 1) and HT-29R cell (Figure 2). HT-29 cells without resistance to irinotecan were more sensitive to simvastatin (IC_50_  115.4 ± 0.14 *μ*M (*r* = 0.98)) or irinotecan (IC_50_  115.4 ± 0.14 *μ*M (*r* = 0.98)) than HT-29R cells with irinotecan resistance (IC_50_  221.9 ± 0.22 *μ*M (*r* = 0.98) and 195.9 ± 0.16 *μ*M (*r* = 0.99)).

### 3.3. Effect of Various Combinations of Simvastatin and Irinotecan in HT-29 and HT-29R Cells

In order to investigate the combination effects of two drugs in colon cancer cells, fixed molar ratio combinations of simvastatin and irinotecan were investigated and especially, the two drugs were treated at various combinations of molar ratios of 4 : 1, 2 : 1, 1 : 1, 1 : 2, and 1 : 4 based on IC_50_ values of two drugs on HT-29 and HT-29R cells in this study (Table 1). The most efficient way for experimental design is to choose the combination drugs at their equipotent ratio (at the ratio of their IC_50_). We serially diluted the mixture (1-fold, 0.5-fold, and 0.25-fold dilution) of these two drugs to obtain a good dosage range.

**Figure 3:**
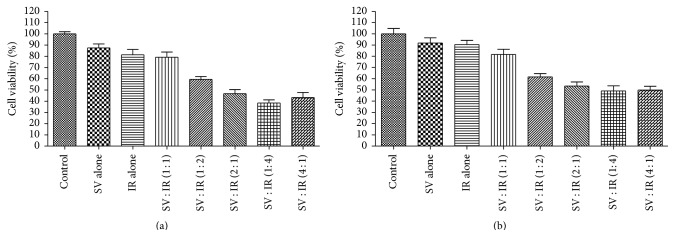
Effect of various fixed ratio combinations of simvastatin and irinotecan on cell viability in HT-29 cells and irinotecan-resistant HT-29 cells. Simvastatin (SV) and irinotecan (IR) were administered at molar ratios of 4 : 1, 2 : 1, 1 : 1, 1 : 2, and 1 : 4 based on IC_50_ values of these two drugs on HT-29 cells and HT-29R cells. Cell proliferation of HT-29 (a) and HT-29R cells (b) at various molar ratios of both drugs was measured by MTT assay. These results showed that combined treatment of irinotecan with simvastatin more potently suppressed cell proliferation of HT-29 cells even with resistance to irinotecan as well as without resistance to irinotecan. The data are expressed as the means ± SD from three independent experiments.

**Figure 4:**
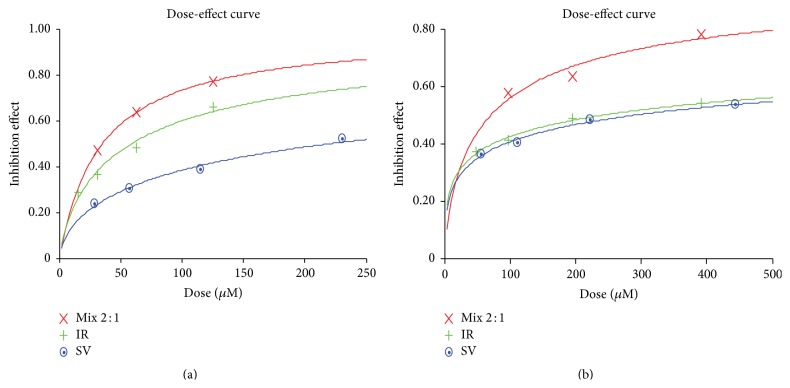
Dose-effect curve of simvastatin and irinotecan in HT-29 cells and HT-29R cells. Combination analysis was done using the method described by Chou and Talalay as described in Section 2. A representative experiment result (repeated at least three times) is reported. Dose-effect curves showed that the combination of simvastatin (SV) and irinotecan (IR) at 2 : 1 ratio is significantly potent rather than SV or IR alone in HT-29 cells (a) and HT-29R cells (b).

MTT assay measured cell proliferation of HT-29 and HT-29R cells at various combinations of molar ratios of both drugs (Figure 3). The measure of a synergistic effect between the two drugs was determined by the CI value derived from the median effect principle described by Chou and Talalay using the software CalcuSyn 3.0.  Figure 4 showed the dose-effect plots of CI against fraction affected for the various combinations of simvastatin and irinotecan in HT-29 and HT-29R cells. It is important to assess whether the drug combination has synergistic effect on maximum eradication of cancer cells; thus, Table 2 showed that the CI values of two drugs were investigated at the various combination ratios. The 2 : 1 molar ratio of simvastatin and irinotecan appeared to be the most promising, with a CI value of 0.34 in HT-29 cells indicating synergy and a CI value of 0.42 in HT-29R cells indicating synergy.

### 3.4. Effect of Simvastatin/Irinotecan at 2 : 1 Molar Ratio on the Apoptosis of Colon Cancer Cells

**Figure 5:**
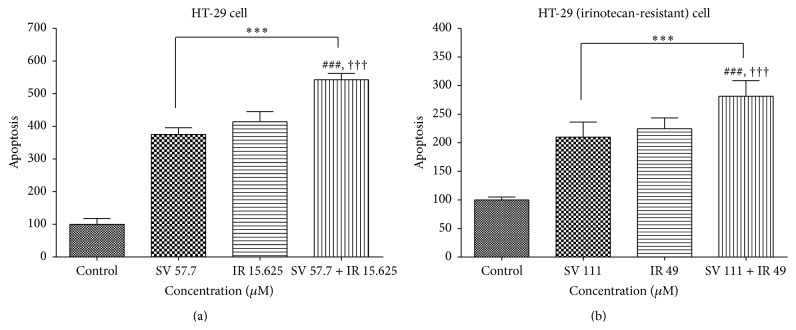
Synergistic effect of simvastatin and irinotecan on cell death in HT-29 cell and irinotecan-resistant HT-29 cells. Cell apoptosis was assessed by the detection of monooligonucleosomes (histone-associated DNA fragments) using an ELISA technique in HT-29 cells and HT-29R cells. (a) HT-29 cells were treated for 48 h with simvastatin (SV) alone, irinotecan (IR) alone, and combination of SV and IR at 2 : 1 molar ratio in which the concentration of SV and IR was 57.7 *μ*M and 15.625 *μ*M, respectively. (b) HT-29R cells were treated for 48 h with SV alone, IR alone, and combination of SV and IR at 2 : 1 molar ratio in which the concentration of SV and IR was 111 *μ*M and 49 *μ*M, respectively. Increased apoptotic responses were evident in combination treatment group of the cells relative to untreated control and single treatment of SV or IR in HT-29 cells and HT-29R cells. The data are expressed as the means ± SD from three independent experiments. ^*∗∗∗*^ *P* < 0.001, compared to untreated control cells. ^*∗∗∗*^ *P* < 0.001, compared to untreated control cells; ^###^ *P* < 0.05, compared to SV alone; ^†††^ *P* < 0.05, compared to IR alone.

**Figure 6:**
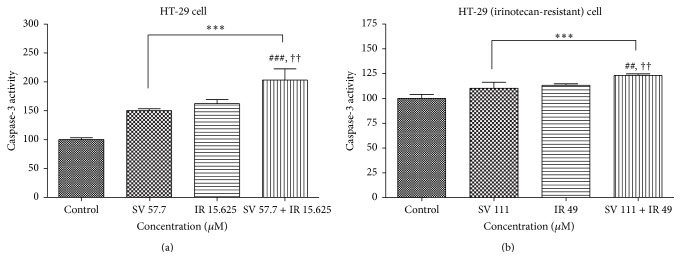
Synergistic effect of simvastatin and irinotecan on caspase-3 activity in HT-29 cell and irinotecan-resistant HT-29 cells. The spectrophotometric assay of caspase-3 activity was carried out as described in Section 2. HT-29 cells were treated for 48 h with simvastatin (SV) alone, irinotecan (IR) alone, and combination of SV and IR at 2 : 1 molar ratio in which the concentration of SV and IR was 57.7 *μ*M and 15.625 *μ*M, respectively (a). Following each drug treatment for 48 h, the spectrofluorometric assay of caspase-3 activity was carried out. The data are expressed as the means ± SD from three independent experiments. ^*∗∗∗*^ *P* < 0.001, compared to untreated control cells; ^###^ *P* < 0.001, compared to SV alone; ^††^ *P* < 0.05, compared to IR alone. HT-29R cells were treated for 48 h with SV alone, IR alone, and combination of SV and IR at 2 : 1 molar ratio in which the concentration of SV and IR was 111 *μ*M and 49 *μ*M, respectively (b). Following each drug treatment for 48 h, caspase-3 activity was determined spectrophotometrically using a multiplate reader (DTX 880 Multimode Detector). Increased caspase-3 activities were evident in combination treatment group of the cells relative to untreated control and single treatment of SV or IR in HT-29 cells (a) and HT-29R cells (b). The data are expressed as the means ± SD from three independent experiments. ^*∗∗∗*^ *P* < 0.001, compared to untreated control cells; ^##^ *P* < 0.05, compared to SV alone; ^††^ *P* < 0.05, compared to IR alone.

In order to investigate the effect of simvastatin and irinotecan combination on the apoptosis of colon cancer cells with or without resistance, we performed cell death detection ELISA tests and caspase-3 activity assay on HT-29 and HT-29R cells treated with simvastatin or irinotecan alone or the combination of simvastatin and irinotecan at 2 : 1 molar ratio. HT-29 cells were treated for 48 h with simvastatin alone, irinotecan alone, and the combination of simvastatin and irinotecan at 2 : 1 molar ratio, respectively. As shown in Figure 5, cell death detected by ELISA test was significantly more increased with the combination treatment of simvastatin and irinotecan than simvastatin or irinotecan alone in HT-29 cells and even HT-29R cells. Caspase-3 is a caspase protein that interacts with caspase-8 and caspase-9. Sequential activation of caspases plays a central role in the cell apoptosis. As shown in Figure 6, activity of caspase-3 was also significantly increased with the combination treatment of simvastatin and irinotecan compared to simvastatin or irinotecan alone in HT-29R cells as well as HT-29 cells.

## 4. Discussion

In the present study, the anticancer activity of simvastatin as a combination drug with irinotecan is compared to that of a commonly used chemotherapeutic drug, irinotecan alone in the colon cancer cell lines, HT-29 cells with or without irinotecan resistance. Our results demonstrated that the combination of simvastatin and irinotecan was more potent than irinotecan or simvastatin alone.

Statins are mainly used to reduce cholesterol levels in patients with cardiovascular or cerebrovascular diseases. Recently some data have shown that statins exert various pleiotropic effects besides their well-known potency to decrease levels of cholesterol, including anticancer or antioxidant effects [16, 17]. Previous studies have shown that statins, such as simvastatin, potently inhibit colon cancer cell proliferation [24]. Others revealed that statin induces cancer cell apoptosis [25] and inhibits tumor-associated angiogenesis [26]. Migration of cancer cells is an important step in the distant metastasis of malignant cells [27]. A recent study has shown that simvastatin potently inhibits CCL17-induced colon cancer cell migration [28]. We also previously demonstrated that simvastatin increases the apoptosis of colon cancer cells and suppresses ERK and Akt via the downregulation of IGF-1R expression and proapoptotic ERK activation and might be beneficial for the treatment of colon cancer. The effect that statins exert on cells depends on many factors, specifically on the structure of the statin and its ability to penetrate cell membranes, time of exposure, and statin concentration. Simvastatin is a hydrophobic statin which means high cytotoxic potential while hydrophilic statin such as pravastatin has little effect on the viability of cell lines in most of proliferation and apoptosis experiments [29–31].

We demonstrated for the first time that the combination of simvastatin and irinotecan can synergistically inhibit colon cancer cell proliferation in HT-29 cells with or without irinotecan resistance. We first established the irinotecan-resistant HT-29 cells as the same method described by previous studies [23]. There was a statistically significant difference between HT-29 and HT-29R cells in terms of IC_50_ measured by each cell line. Combination indexes for various fixed ratio combinations of simvastatin and irinotecan revealed that 2 : 1 molar ratio is the most promising one indicating the synergistic effect on HT-29 cells with or without irinotecan resistance. Our study showed that combination treatment with simvastatin and irinotecan at 2 : 1 molar ratio more potently induces the apoptosis of colon cancer cells with or without irinotecan resistance. This result suggests that simvastatin may be of beneficial role in the treatment of colon cancers, especially in order to overcome irinotecan-resistance.

Irinotecan (CPT-11), a semisynthetic water-soluble derivative of camptothecin, is widely used for the treatment of metastatic colon cancer in first- and second-line therapies [32]. Drug resistance to chemotherapeutic agents frequently results in treatment failure in CRC patients. The cellular mechanisms associated with this resistance lead to making a significant improvement in terms of the treatment of CRC. Cellular resistance to camptothecin derivatives results from a decrease in drug accumulation within cells, change in the structure or location of topoisomerase I, alteration in the cellular response to the drug-DNA-enzyme ternary complex formation, or increased glucuronidation of SN-38, ultimately causing an inactivation of chemotherapeutic drugs [33]. Members of the ATP-binding cassette (ABC) transporters, MDR1 (ABCB1) and MRP1 (ABCC1), confer resistance to chemotherapeutic medicines by active drug efflux [34, 35]. One study showed that exposure of the human colorectal cancer cell line HCT116 to SN-38 induces the expression of ABCG2 protein, and overexpression of ABCG2 suggests high levels of resistance to SN-38 [23]. A recent study showed that prolonged exposure to SN-38/irinotecan leads to some permanent modifications of cell cycle dynamics in vitro [36]. SN-38 resistant HT-29/HCT116 cell lines had a prolonged generation doubling time which was associated with doubling of the fraction of cells in G2/M and a decreased proportion of S phase cells [36].

In this study, the results of IC_50_ for these two drugs on HT-29 cells with irinotecan resistance showed that HT-29 cells with irinotecan resistance seemed to obtain cross-resistance to simvastatin. Despite cross-resistance to simvastatin, HT-29 cells with irinotecan resistance showed synergistic effect of simvastatin and irinotecan in this study. One of the possible mechanisms of a synergistic effect between statin and irinotecan in irinotecan-resistant colon cancer cells is suggested that depletion of cholesterol by simvastatin inhibits the activity of ABCG2 receptors, which is important in irinotecan resistance. Cholesterol is able to alter structures and function of membrane-bound proteins, including ABC transporters. Recently some studies have shown that membrane cholesterol plays an important role in regulation of ABCB1 and ABCG2 activity, and lipid-lowering drugs might be able to overcome drug resistance with inhibiting ABCG2/ABCB1 transporters through cholesterol depletion [37–39]. The other possible mechanisms of synergistic effect of statin and irinotecan in colon cancer cells are suggested that statins can augment the chemosensitivity of colorectal cancer cells inducing epigenetic reprogramming and reducing colorectal cancer cell stemness via the bone morphogenetic protein pathway [22].

In this study, we used irinotecan (CPT-11) rather than SN-38, an active metabolite of irinotecan. Irinotecan could be used for the treatment on colon cancer cells for the purpose of apoptosis study or drug combination study [40, 41]. However SN-38 would have shown better cytotoxic results than irinotecan as a limitation of our study.

Taken together, this study shows for the first time that simvastatin and irinotecan have a synergistic effect to improve eliminating colon cancer cells with or without irinotecan resistance in vitro. This result suggests that simvastatin has some therapeutic potential to overcome irinotecan-resistant colon cancer. Future work will more fully explore the mechanisms and role of statins in the treatment of colon cancer.

## Figures and Tables

**Table 1:** Drug concentrations of simvastatin and irinotecan at various molar ratio based on IC_50_ value of each drug in HT-29 cells and irinotecan-resistant HT-29 cells.

Molar ratio	HT-29		Irinotecan-resistant HT-29	
	Simvastatin (*μ*M)	Irinotecan (*μ*M)	Simvastatin (*μ*M)	Irinotecan (*μ*M)
1	28.85	15.625	55.5	49
2	57.7	31.25	111.0	98
4	115.4	62.5	221.9	195.93

*R* ^2^	0.98	0.98	0.98	0.99

IC_50_	115.4 ± 0.14 *μ*M	62.5 ± 0.18 *μ*M	221.9 ± 0.22 *μ*M	195.9 ± 0.16 *μ*M

**Table 2:** Combination index values of simvastatin and irinotecan in HT-29 and HT-29R cells.

Drug combination	Ratio	Combination index of HT-29			Combination index of HT-29R		
		IC_50_	IC_75_	IC_90_	IC_50_	IC_75_	IC_90_
Simvastatin : irinotecan	1 : 1	0.70	0.40	0.24	0.72	0.41	0.24
	1 : 2	0.68	0.48	0.34	0.43	0.22	0.01
	2 : 1	0.34	0.21	0.14	0.42	0.08	0.01
	1 : 4	0.83	0.60	0.43	0.46	0.07	0.01
	4 : 1	0.59	0.26	0.12	0.82	0.39	0.31

CI < 1 synergistic effect, CI = 1 additive effect, and CI > 1 antagonistic effect.

## References

[1] Jemal A., Siegel R., Ward E., Hao Y., Xu J., Thun M. J. (2009). Cancer statistics, 2009. *CA: A Cancer Journal for Clinicians*.

[2] Siegel R., Naishadham D., Jemal A. (2013). Cancer statistics, 2013. *CA Cancer Journal for Clinicians*.

[3] Shin A., Kim K.-Z., Jung K.-W. (2012). Increasing trend of colorectal cancer incidence in Korea, 1999–2009. *Cancer Research and Treatment*.

[4] Park S. H., Song C. W., Kim Y. B. (2014). Clinicopathological characteristics of colon cancer diagnosed at primary health care institutions. *Intestinal Research*.

[5] O'Connell J. B., Maggard M. A., Ko C. Y. (2004). Colon cancer survival rates with the new American Joint Committee on Cancer sixth edition staging. *Journal of the National Cancer Institute*.

[6] Boghossian S., Hawash A. (2012). Chemoprevention in colorectal cancer—where we stand and what we have learned from twenty year's experience. *Surgeon*.

[7] Zhang N., Yin Y., Xu S.-J., Chen W.-S. (2008). 5-Fluorouracil: mechanisms of resistance and reversal strategies. *Molecules*.

[8] Rothenberg M. L. (1997). Topoisomerase I inhibitors: review and update. *Annals of Oncology*.

[9] Pizzolato J. F., Saltz L. B. (2003). The camptothecins. *Lancet*.

[10] Fuchs C., Mitchell E. P., Hoff P. M. (2006). Irinotecan in the treatment of colorectal cancer. *Cancer Treatment Reviews*.

[11] Litvak D. A., Bilchik A. J., Cabot M. C. (2003). Modulators of ceramide metabolism sensitize colorectal cancer cells to chemotherapy: a novel treatment strategy. *Journal of Gastrointestinal Surgery*.

[12] Van Cutsem E., Blijham G. H. (1999). Irinotecan versus infusional 5- fluorouracil: a phase III study in metastatic colorectal cancer following failure on first-line 5-fluorouracil. V302 Study Group. *Seminars in Oncology*.

[13] Liu Y., Tang W., Wang J. (2014). Association between statin use and colorectal cancer risk: a meta-analysis of 42 studies. *Cancer Causes and Control*.

[14] Poynter J. N., Gruber S. B., Higgins P. D. R. (2005). Statins and the risk of colorectal cancer. *The New England Journal of Medicine*.

[15] Bardou M., Barkun A., Martel M. (2010). Effect of statin therapy on colorectal cancer. *Gut*.

[16] Blais L., Desgagné A., LeLorier J. (2000). 3-Hydroxy-3-methylglutaryl coenzyme a reductase inhibitors and the risk of cancer: a nested case-control study. *Archives of Internal Medicine*.

[17] Graaf M. R., Beiderbeck A. B., Egberts A. C. G., Richel D. J., Guchelaar H.-J. (2004). The risk of cancer in users of statins. *Journal of Clinical Oncology*.

[18] Cho S. J., Kim J. S., Kim J. M., Lee J. Y., Jung H. C., Song I. S. (2008). Simvastatin induces apoptosis in human colon cancer cells and in tumor xenografts and attenuates colitis-associated colon cancer in mice. *International Journal of Cancer*.

[19] Ukomadu C., Dutta A. (2003). p21-dependent inhibition of colon cancer cell growth by mevastatin is independent of inhibition of G_1_ cyclin-dependent kinases. *The Journal of Biological Chemistry*.

[20] Wächtershäuser A., Akoglu B., Stein J. (2001). HMG-CoA reductase inhibitor mevastatin enhances the growth ihibitory effect of butyrate in the colorectal carcinoma cell line Caco-2. *Carcinogenesis*.

[21] Agarwal B., Halmos B., Feoktistov A. S. (2002). Mechanism of lovastatin-induced apoptosis in intestinal epithelial cells. *Carcinogenesis*.

[22] Kodach L. L., Jacobs R. J., Voorneveld P. W. (2011). Statins augment the chemosensitivity of colorectal cancer cells inducing epigenetic reprogramming and reducing colorectal cancer cell ‘stemness’ via the bone morphogenetic protein pathway. *Gut*.

[23] Candeil L., Gourdier I., Peyron D. (2004). ABCG2 overexpression in colon cancer cells resistant to SN38 and in irinotecan-treated metastases. *International Journal of Cancer*.

[24] Bergman M., Salman H., Djaldetti M., Bessler H. (2011). Statins as modulators of colon cancer cells induced cytokine secretion by human PBMC. *Vascular Pharmacology*.

[25] Lee J., Lee I., Han B. (2011). Effect of simvastatin on cetuximab resistance in human colorectal cancer with KRAS mutations. *Journal of the National Cancer Institute*.

[26] Park H.-J., Kong D., Iruela-Arispe L., Begley U., Tang D., Galper J. B. (2002). 3-Hydroxy-3-methylglutaryl coenzyme A reductase inhibitors interfere with angiogenesis by inhibiting the geranylgeranylation of RhoA. *Circulation Research*.

[27] Vega F. M., Ridley A. J. (2008). Rho GTPases in cancer cell biology. *FEBS Letters*.

[28] Al-Haidari A. A., Syk I., Thorlacius H. (2014). HMG-CoA reductase regulates CCL17-induced colon cancer cell migration via geranylgeranylation and RhoA activation. *Biochemical and Biophysical Research Communications*.

[29] Sivaprasad U., Abbas T., Dutta A. (2006). Differential efficacy of 3-hydroxy-3-methylglutaryl CoA reductase inhibitors on the cell cycle of prostate cancer cells. *Molecular Cancer Therapeutics*.

[30] Cafforio P., Dammacco F., Gernone A., Silvestris F. (2005). Statins activate the mitochondrial pathway of apoptosis in human lymphoblasts and myeloma cells. *Carcinogenesis*.

[31] Matusewicz L., Meissner J., Toporkiewicz M., Sikorski A. F. (2015). The effect of statins on cancer cells-review. *Tumor Biology*.

[32] Rougier P., Mitry E. (2000). Colorectal cancer chemotherapy: irinotecan. *Seminars in Oncology*.

[33] Takahashi T., Fujiwara Y., Yamakido M., Katoh O., Watanabe H., Mackenzie P. I. (1997). The role of glucuronidation in 7-ethyl-10-hydroxycamptothecin resistance in vitro. *Japanese Journal of Cancer Research*.

[34] Gottesman M. M., Fojo T., Bates S. E. (2002). Multidrug resistance in cancer: role of ATP-dependent transporters. *Nature Reviews Cancer*.

[35] Litman T., Druley T. E., Stein W. D., Bates S. E. (2001). From MDR to MXR: new understanding of multidrug resistance systems, their properties and clinical significance. *Cellular and Molecular Life Sciences*.

[36] Petitprez A., Poindessous V., Ouaret D. (2013). Acquired irinotecan resistance is accompanied by stable modifications of cell cycle dynamics independent of MSI status. *International Journal of Oncology*.

[37] Glodkowska-Mrowka E., Mrowka P., Basak G. W. (2014). Statins inhibit ABCB1 and ABCG2 drug transporter activity in chronic myeloid leukemia cells and potentiate antileukemic effects of imatinib. *Experimental Hematology*.

[38] Telbisz Á., Müller M., Özvegy-Laczka C. (2007). Membrane cholesterol selectively modulates the activity of the human ABCG2 multidrug transporter. *Biochimica et Biophysica Acta (BBA)—Biomembranes*.

[39] Eckford P. D. W., Sharom F. J. (2008). Interaction of the P-glycoprotein multidrug efflux pump with cholesterol: effects on ATPase activity, drug binding and transport. *Biochemistry*.

[40] Rudolf E., Kralova V., Rudolf K., John S. (2013). The role of p38 in irinotecan-induced DNA damage and apoptosis of colon cancer cells. *Mutation Research*.

[41] Ling L.-U., Lin H., Tan K.-B., Chiu G. N. C. (2009). The role of protein kinase C in the synergistic interaction of safingol and irinotecan in colon cancer cells. *International Journal of Oncology*.

